# Artificial Intelligence-Powered High-Content Analysis: Methodologies and Applications in Bioactive Compound Discovery from Natural Sources

**DOI:** 10.3390/molecules31173075

**Published:** 2026-09-01

**Authors:** Dejin Xun, Zuyong Zhang, Han Wang, Yingchao Wang, Xiaohui Fan, Yi Wang

**Affiliations:** 1 Pharmaceutical Informatics Institute, School of Pharmacy, Zhejiang University, Hangzhou 310058, China; xundejin@zju.edu.cn (D.X.); zzynwk@126.com (Z.Z.); wangyingchao@zju.edu.cn (Y.W.); 2 National Key Laboratory of Chinese Medicine Modernization, Innovation Center of Yangtze River Delta, Zhejiang University, Jiaxing 314100, China; 3 Department of Dermatology, Hangzhou Third People’s Hospital, 38 Xihu Avenue, Hangzhou 310009, China; 4 School of Chemistry and Chemical Engineering, Shanghai University of Engineering Science, 333 Longteng Road, Shanghai 201620, China; wanghan@sues.edu.cn; 5 Innovation Institute for Artificial Intelligence in Medicine of Zhejiang University, Hangzhou 310018, China

**Keywords:** high-content analysis, deep learning, natural products, image analysis, phenotypic drug discovery

## Abstract

High-content screening (HCS) is a useful phenotypic drug discovery technology that combines automated microscopy, image analysis, and high-throughput experimentation to comprehensively characterize biological responses to diverse perturbations. This review summarizes the methodological fundamentals of high-content analysis, including image preprocessing, cell segmentation, feature processing, and downstream analysis, as well as the diverse phenotypic datasets generated from different biological models, perturbation strategies, and staining approaches. Recent advances in artificial intelligence, particularly deep learning, have improved cell segmentation, image representation learning, and phenotypic profiling, enabling more accurate and scalable analysis of HCS data. We further highlight emerging applications of AI-powered HCS in pharmaceutical research, with a particular focus on the discovery of bioactive compounds from natural sources. Finally, we discuss current challenges and future perspectives, including the construction of large-scale phenotypic databases, the integration of AI throughout the screening workflow, and the development of intelligent screening platforms. These advances are expected to accelerate phenotype-driven drug discovery and promote innovation in natural product research.

## 1. Introduction

High-content screening is an image-based phenotypic screening technology that combines automated microscopy, fluorescent labeling, and quantitative image analysis to systematically characterize cellular responses to genetic, chemical, or environmental perturbations [1,2,3,4,5]. By simultaneously measuring multiple morphological, spatial, and functional features, HCS provides a multidimensional representation of cellular phenotypes that extends far beyond conventional endpoint assays. Modern HCS platforms integrate high-throughput imaging systems with advanced computational pipelines, enabling the acquisition and analysis of millions of cellular images across diverse biological models [6,7,8]. As a result, HCS has become an indispensable tool in numerous fields, including drug discovery, functional genomics, toxicology, and rare disease research [9,10,11,12,13,14,15].

Unlike traditional target-based screening approaches, which typically focus on a single molecular target or biochemical readout, HCS can capture holistic cellular responses and allow the identification of bioactive compounds through phenotypic changes. This capability is particularly valuable for natural product research. Natural products and herbal medicines contain structurally diverse molecules that often exert their biological activities through multiple targets and pathways. Such complexity presents significant challenges for conventional screening methods, which frequently fail to capture the full spectrum of biological activities [16,17,18,19,20]. In contrast, HCS enables unbiased phenotypic profiling and provides rich cellular information, including changes in morphology, organelle organization, signaling pathways, and cellular functions, thereby facilitating the discovery of bioactive compounds.

The development of HCS can be traced back to the emergence of automated fluorescence microscopy and image analysis technologies in the late 1990s and early 2000s. Early HCS studies relied heavily on manually designed image features and classical machine learning algorithms for phenotype classification. While these approaches significantly improved the throughput of phenotypic screening, their performance was constrained by limited feature representation and the complexity of biological images. Over the past decade, rapid advances in artificial intelligence (AI), particularly deep learning, have transformed the field. Convolutional neural networks, vision transformers, self-supervised learning frameworks, and generalist models have demonstrated unprecedented capabilities in image segmentation, feature extraction, and phenotype classification [21,22,23,24,25]. These advances have enabled the extraction of biologically meaningful information directly from raw microscopy images, reducing the dependence on handcrafted features and improving analytical accuracy and scalability.

The integration of AI with HCS has been especially transformative for the discovery of bioactive compounds from natural sources. Natural product libraries and herbal extracts often contain thousands of chemically diverse constituents with unknown biological activities. AI-driven HCS provides a useful platform for systematically interrogating these complex resources, prioritizing active fractions, predicting mechanisms of action, identifying potential targets, and uncovering novel therapeutic opportunities [6,7,8]. Despite these advances, several challenges remain. The high dimensionality and heterogeneity of imaging data, batch effects [26,27] across experimental platforms, limited availability of annotated datasets, and the interpretability of AI models continue to hinder broader adoption. In addition, the complexity of natural product mixtures presents unique difficulties for data analysis, activity attribution, and experimental standardization. Currently, HCS has attracted increasing attention in natural product research. A recent review [28] has summarized the applications of HCS technology in traditional Chinese medicine (TCM) research, with a particular focus on the implementation of HCS studies, optimization of experimental parameters, and major application areas. However, it does not provide a comprehensive discussion of HCA from a computational perspective or cover recent advances in AI-based HCA. In this review, we therefore focus on the methodological advances in HCA and the applications of AI-based HCA in bioactive compound discovery. Otherwise, we also primarily focus on traditional Chinese medicine (TCM) and plant-derived natural products, which represent important sources of bioactive compounds. Although natural sources also include microbial, fungal, marine, and animal sources, these areas are beyond the scope of our expertise and are therefore not covered in this review.

This review provides a comprehensive overview of recent advances in AI-driven high-content analysis (HCA). Section 2 outlines the methodological fundamentals of HCA, diverse phenotypic data generated by HCS experiments, and emerging AI-powered analytical approaches. Section 3 highlights recent advances in deep learning-based intelligent HCS and its applications in the discovery of bioactive compounds from natural sources. Finally, Section 4 discusses the current challenges and future perspectives of AI-powered high-content screening in natural product research, with a focus on phenotypic database construction, AI integration across the screening workflow, and the development of intelligent screening platforms.

## 2. Methodological Fundamentals of HCA

### 2.1. Typical High-Content Analysis

**Figure 1:**
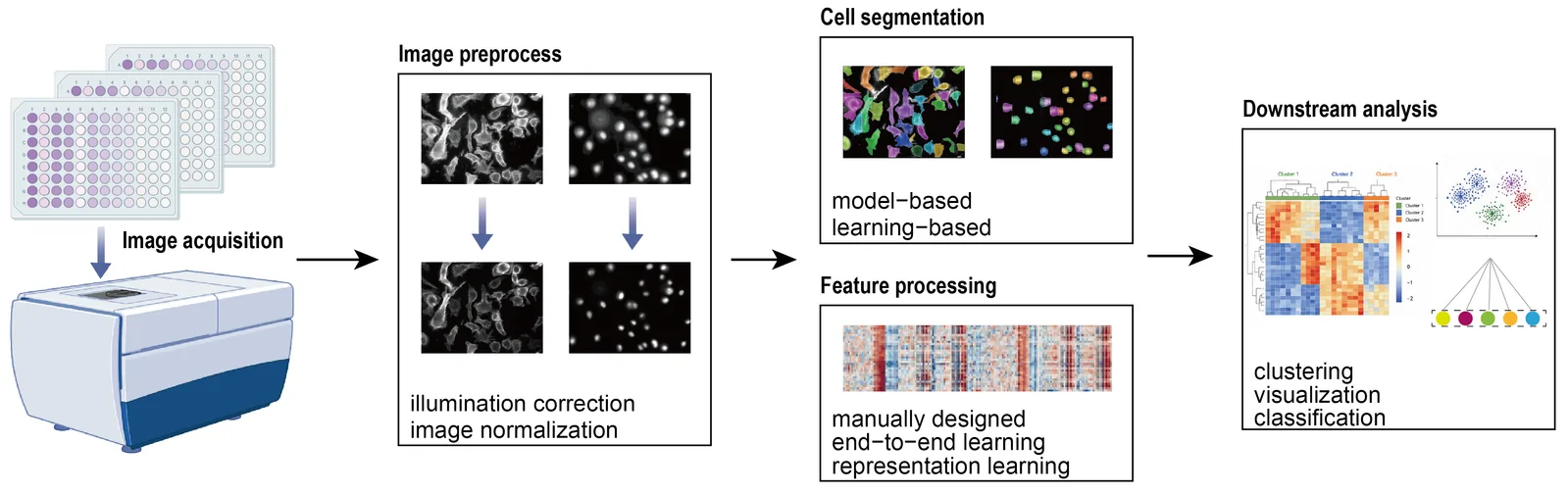
Overview of the high-content analysis workflow.

High-content analysis refers to the process of converting digital microscopy images into quantitative measurements for each experimental perturbation and performing downstream analyses on these measurements. The resulting data can be organized into a matrix in which each row represents a perturbation and each column corresponds to a feature extracted from the observed phenotype. A typical HCA workflow (Figure 1) consists of four major steps: image preprocessing, cell segmentation, feature processing, and downstream analysis.

The first step is image preprocessing. Due to variations in illumination sources and vignetting effects commonly observed at image edges, acquired images often exhibit non-uniform illumination, resulting in intensity fluctuations of approximately 10–30%, which can affect downstream analyses [29]. Therefore, illumination correction is routinely performed in traditional image analysis pipelines. Illumination correction methods can generally be categorized into three groups. Prospective methods construct correction models using reference images acquired during microscopy imaging and therefore require careful calibration during image acquisition. Retrospective single-image methods generate an independent correction model for each image based solely on the image itself. Although these methods reduce the burden of image acquisition, they may compromise comparability across images. In contrast, retrospective multi-image methods build correction models using all images collected within an experiment, resulting in more robust correction and consequently becoming the most widely adopted approach in biological laboratories. In addition to illumination correction, normalization and standardization are frequently applied. Normalization transforms pixel intensities into a predefined range, typically between 0 and 1. For example, min-max normalization rescales each pixel value by subtracting the minimum pixel intensity and dividing by the intensity range of the image. This procedure reduces variations across images and improves comparability between datasets acquired under different conditions. Standardization, on the other hand, subtracts the mean pixel value and divides by the standard deviation, producing a normalized distribution with zero mean and unit variance. This process mitigates global intensity shifts, balances feature scales, and prevents specific features from disproportionately influencing model training.

The second step is cell segmentation. By identifying and isolating individual cellular instances within an image, cell segmentation enables quantitative analysis at single-cell resolution. Fundamentally, segmentation is a pixel-level classification task. Existing segmentation methods can generally be divided into two categories. The first category comprises model-based methods, which rely on prior knowledge and manual parameter optimization based on visual inspection of segmentation results. Parameters may include expected object size and shape or image histogram distributions. Most model-based methods belong to the category of binary semantic segmentation and generate a binary mask corresponding to the original image, where pixels are classified as either foreground (cell) or background. Common approaches include threshold-based methods such as Otsu thresholding, edge-based methods such as edge detection algorithms, region-based methods including region growing and watershed segmentation, and graph-based methods such as GraphCut [30]. The second category consists of machine learning-based methods. These approaches utilize paired training datasets containing raw images and manually annotated masks to learn segmentation models automatically. Compared with model-based methods, machine learning approaches require fewer manually defined parameters and generally achieve superior performance in complex imaging scenarios [31]. However, early machine learning models often exhibited limited generalizability, requiring extensive manual annotation and programming expertise when applied to new datasets. In contrast, many model-based methods have been integrated into user-friendly software packages such as CellProfiler 3.0 [32] and ImageJ2, making them accessible to researchers without computational backgrounds. Consequently, these approaches have remained widely used in many laboratories.

The third step is feature processing. Features represent quantitative measurements extracted from images and contain information describing cellular phenotypes. Feature processing consists of two key procedures: feature extraction and feature aggregation. Feature extraction strategies can be broadly classified into three categories. The first involves the direct measurement of manually designed features. Building upon previous studies, Carpenter et al. developed CellProfiler, which enables extraction of a wide variety of image features through an intuitive graphical interface. These features can generally be divided into three groups. The first group characterizes full images, including image quality metrics, overall fluorescence intensity, and cell counts. The second group quantifies individual cells, including measurements such as fluorescence intensity, area, size, and circularity. The third group captures microenvironmental information, including neighboring-cell relationships and organelle colocalization patterns. The second strategy integrates feature extraction directly into downstream tasks through end-to-end deep learning frameworks. For example, Chen et al. [33] trained a classification model directly on raw microscopy images to distinguish damaged, healthy, and irrelevant cells, with feature extraction implicitly learned within a VGG-based architecture. The third strategy is based on representation learning [34], in which feature extraction is decoupled from downstream tasks. Rather than relying on manually designed descriptors, representation learning approaches learn generalizable feature representations directly from large-scale datasets. Following feature extraction, the features obtained from individual images must be aggregated. For example, in a typical HCS experiment, a single perturbation may correspond to one well, with four fields of view imaged per well. Under a single-cell analysis framework, features extracted from individual cells are first aggregated into image-level representations, which are then combined across multiple fields of view to generate perturbation-level profiles. Common aggregation strategies include mean profiling, median profiling, and Kolmogorov–Smirnov profiling.

The final step is downstream analysis. Through visualization techniques and machine-learning approaches, extracted phenotypic features can be interpreted and validated to reveal biologically meaningful patterns and relationships. Three major downstream analysis strategies are commonly employed. The first is clustering, which groups perturbations with similar phenotypic profiles in an unsupervised manner. Clustering can not only validate known biological relationships but also uncover previously unrecognized associations among treatments. A widely used method is hierarchical clustering [35], which visualizes similarity matrices as heatmaps and facilitates the identification of patterns across dozens or even hundreds of samples. The second strategy involves dimensionality reduction and visualization. High-dimensional phenotypic features are projected into two- or three-dimensional spaces to facilitate interpretation of the underlying feature distributions. Common dimensionality-reduction methods include principal component analysis (PCA), t-distributed stochastic neighbor embedding (t-SNE) [36], and uniform manifold approximation and projection (UMAP) [37]. The third strategy is classification. Supervised machine-learning models are trained using annotated datasets and subsequently applied to predict phenotypic outcomes for previously unseen perturbations [5]. Commonly used classifiers include support vector machines, random forests, and neural networks.

It should be noted that these steps are not universally required in every HCA workflow and can be modified according to specific research objectives. For example, improvements in imaging quality and algorithmic performance have reduced the necessity of illumination correction in some applications. Similarly, when datasets become extremely large and cell segmentation imposes substantial computational and economic costs, feature extraction can be performed directly on whole images without explicit segmentation. Moreover, morphological similarity may often indicate similar biological effects or phenotypic responses; however, the underlying molecular mechanisms require further experimental validation.

### 2.2. Diverse Phenotypic Data Generated by HCS

**Figure 2:**
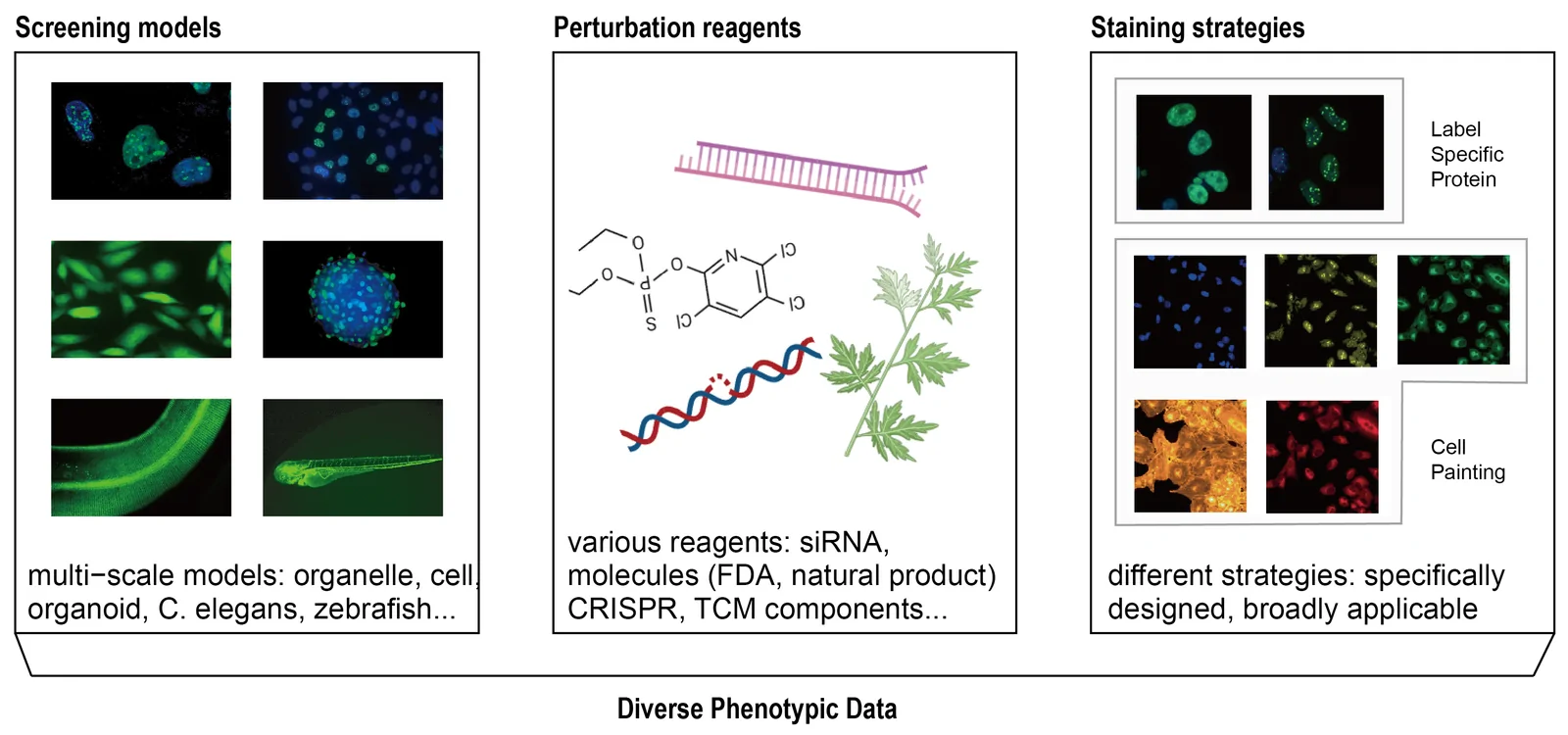
Diversity of phenotypic data in HCA.

Due to the complexity of the screening workflow, HCS generates highly diverse and complex phenotypic datasets. This diversity is mainly reflected in three aspects: biological models, perturbation reagents, and staining strategies (Figure 2). First, a wide variety of screening models can be employed, spanning multiple biological scales from microscopic cellular systems to mesoscopic tissues and even whole organisms. The most commonly used model is the conventional two-dimensional (2D) cell culture system because of its simplicity and convenience. However, accumulating evidence suggests that cellular responses observed in 2D cultures may differ from those observed in native tissues in vivo [38]. To address this limitation, more physiologically relevant biomimetic models have been developed. For example, micropatterning technologies can control the alignment of cardiomyocytes on engineered substrates, promoting coordinated mechanical contraction-relaxation cycles and electrophysiological activity that more closely resemble native cardiac tissues [39]. More recently, organoid technologies [40,41,42,43] have emerged as useful experimental platforms. Compared with traditional cell culture models, organoids possess unique three-dimensional architectures and cell-type-specific organization, enabling them to recapitulate physiological functions and responses that closely resemble those observed in vivo. Consequently, organoid-based models have been increasingly incorporated into HCS workflows. In addition, small model organisms such as *C. elegans* [44,45] and zebrafish [46,47] can be utilized for phenotypic screening, allowing biological responses to be monitored at the level of an intact organism.

Second, HCS can employ a diverse range of perturbation reagents, which may be selected either randomly or specifically designed to interrogate particular biological functions. Depending on the experimental design, different types of perturbations can be compared to explore their phenotypic effects. Perturbation libraries may consist of small molecules, including FDA-approved drug collections, natural product libraries, or compound libraries targeting specific diseases or signaling pathways. Alternatively, genetic perturbations can be introduced using siRNA libraries, which reduce gene expression through mRNA degradation or gene silencing, or CRISPR libraries [48], which exploit CRISPR-Cas9-mediated genome editing to induce targeted genetic modifications. Other perturbation resources, such as TCM component libraries and peptide libraries, have also been incorporated into HCS studies, further expanding the scope of phenotypic discovery.

Third, staining strategies are highly diverse and are largely determined by the objectives of the screening campaign. On one hand, fluorescence labeling can be specifically designed to monitor particular biological mechanisms. For example, the DNA damage response protein 53BP1 exhibits a diffuse nuclear distribution under normal conditions but accumulates at sites of DNA double-strand breaks following genotoxic stress. Based on this phenomenon, fluorescent probes targeting 53BP1 have been developed to quantitatively evaluate DNA damage induced by different perturbations [33]. On the other hand, broadly applicable staining panels can be employed to monitor a wide range of cellular processes. Such assays enable the characterization of diverse biological activities, including cell cycle progression, cell adhesion and migration, cellular metabolism, and organelle-specific phenotypic alterations. By combining multiple fluorescent markers within a single experiment, HCS can generate comprehensive phenotypic profiles that capture diverse aspects of cellular behavior.

Together, the diversity of biological models, perturbation reagents, and staining strategies enables HCS to generate rich and multidimensional phenotypic datasets. While this complexity greatly enhances the biological information content of HCS experiments, it also poses substantial challenges for data analysis and interpretation, thereby driving the development of increasingly sophisticated computational and AI-based analytical methods.

### 2.3. Artificial Intelligence-Powered High-Content Analysis

Deep learning employs multi-layer neural network architectures composed of successive layers of trainable parameters to learn hierarchical representations from data. Such deep neural networks (DNNs) typically use differentiable nonlinear activation functions and are trained end-to-end using backpropagation and gradient-based optimization algorithms. Through successive nonlinear transformations, DNNs can automatically extract increasingly abstract feature representations with varying degrees of selectivity and invariance, enabling them to capture complex patterns in input data while reducing reliance on manually engineered features. In recent years, deep learning has transformed HCA, particularly in two key areas: cell segmentation and microscopy image feature extraction.

**Figure 3:**
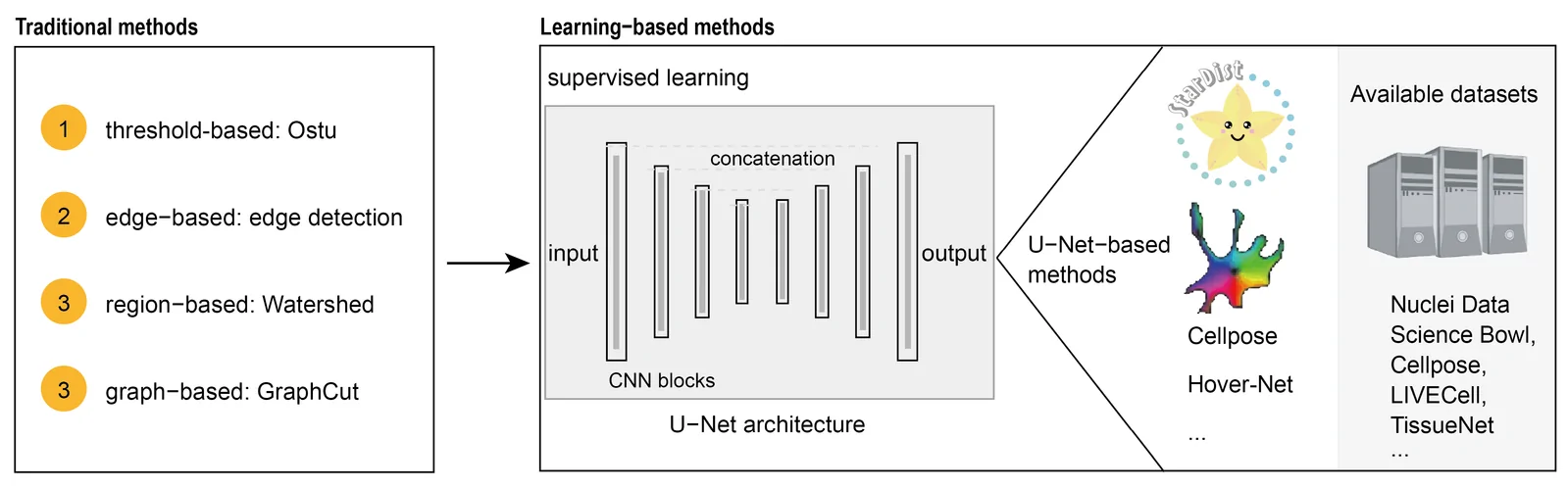
AI-powered cell segmentation methods.

In the deep learning era, the development of cell segmentation algorithms (Figure 3) has largely followed two directions. The first focuses on achieving state-of-the-art performance on specific datasets. In 2015, Ronneberger et al. [21] introduced U-Net, a novel convolutional neural network architecture specifically designed for biomedical image segmentation. Evaluations on electron microscopy and transmitted-light microscopy datasets demonstrated that U-Net significantly outperformed previous sliding-window-based convolutional neural network approaches. Building upon U-Net, Schmidt et al. developed StarDist [49], which represents nuclei as star-convex polygons (see [49] for methodological details) and effectively addresses segmentation errors caused by overlapping or touching cells. Compared with conventional U-Net architectures, StarDist exhibited superior performance in densely packed cellular environments. Subsequently, Graham et al. proposed Hover-Net [50], a deep neural network that encodes instance information using horizontal and vertical distances from nuclear pixels to their corresponding centroids (see [50] for methodological details). This strategy enabled accurate segmentation of clustered nuclei with highly heterogeneous morphologies in hematoxylin and eosin (H&E)-stained histopathology images. The second direction aims to develop general-purpose segmentation methods capable of performing robustly across diverse datasets. In 2021, Stringer et al. developed Cellpose [25], a generalist segmentation framework extending beyond nuclei to more morphologically complex cellular structures. Cellpose was trained on a highly diverse collection of microscopy images and introduced a novel instance representation based on simulated diffusion dynamics (see [25] for methodological details). Specifically, the model was trained to predict the horizontal and vertical gradients of a flow field that encodes cellular topology. Experimental results demonstrated that Cellpose substantially outperformed StarDist while maintaining strong performance across diverse imaging modalities and cell types. Many excellent studies have built upon Cellpose and adapted it to HCS image datasets [51,52]. Otherwise, researchers developed general-purpose models for phase-contrast microscopy and tissue imaging [53,54]. Building upon these advances, the Stringer group continuously refined the Cellpose series [55,56], making substantial contributions to the cell segmentation community. These developments have greatly improved the robustness and accessibility of cell segmentation and have facilitated the widespread adoption of deep learning in HCA workflows.

**Figure 4:**
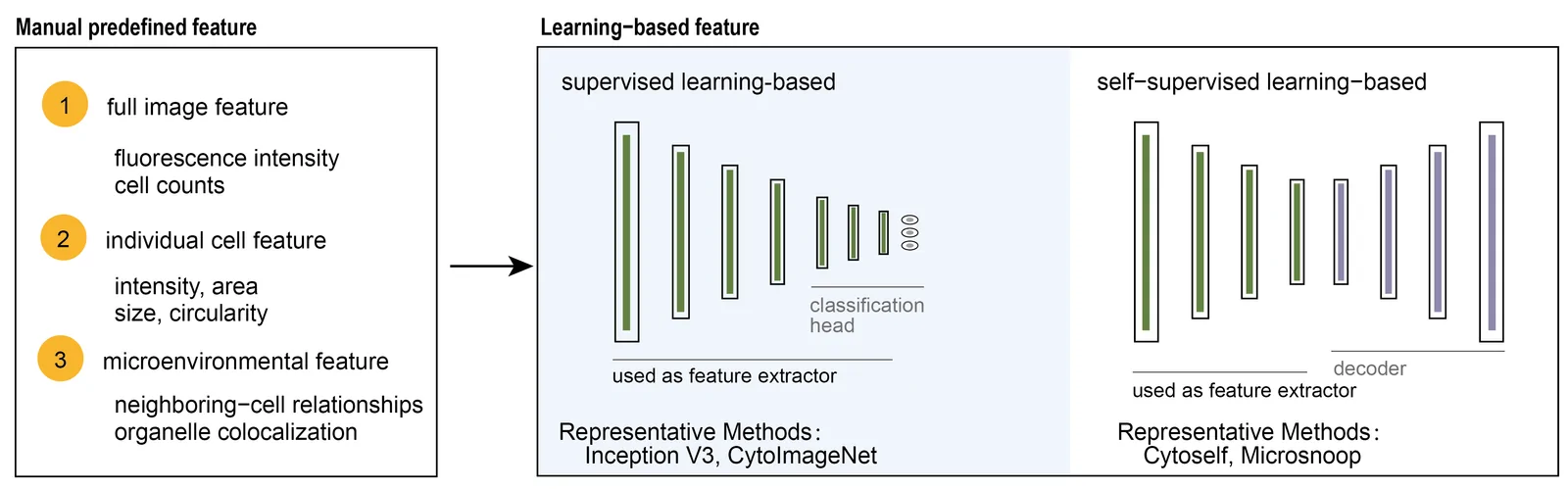
AI-powered microscopy image feature extraction methods.

In addition to segmentation, image feature representation has become another major focus of AI-driven HCA (Figure 4). Early studies primarily focused on dataset-specific representation learning. Pawlowski et al. [57] investigated whether convolutional neural networks pretrained on ImageNet could be directly applied to microscopy image feature extraction. By evaluating multiple architectures, they demonstrated that Inception V3 achieved the best balance between accuracy and computational efficiency on the BBBC021 benchmark dataset. Subsequently, Perakis et al. [58] utilized contrastive learning to generate single-cell representations without requiring manual annotations. Their approach improved mechanism-of-action prediction on the BBBC021 dataset and, for the first time, enabled an unsupervised representation learning method to match the performance of the best supervised approaches available at that time. Around the same period, Hua et al. [59] constructed a microscopy image classification task analogous to ImageNet to learn representations of microscopic images, termed CytoImageNet, which contains approximately 890,000 images spanning 894 categories. However, this approach did not achieve superior performance. Currently, state-of-the-art approaches have increasingly adopted self-supervised learning. In 2022, Kobayashi et al. introduced Cytoself [60], a self-supervised framework built upon the assumption that proteins with similar localization patterns should exhibit similar image representations. By pretraining on 24,382 protein images from the OpenCell database without manual annotation, Cytoself successfully generated high-resolution protein localization maps and demonstrated the power of self-supervised learning for biological image analysis. Another important challenge in HCA is the presence of batch effects. Technical variations arising from differences in experimental batches often degrade model performance. To address this issue, Lin et al. [27] proposed Batch Effects Normalization (BEN), a training strategy that aligns the concept of biological experimental batches with mini-batches used in deep learning optimization. Under this framework, each training mini-batch is sampled exclusively from a single experimental batch. BEN significantly improved representation learning performance and achieved state-of-the-art results on the RxRx1-Wilds benchmark dataset, highlighting the importance of explicitly accounting for experimental variation during model training. In 2024, our team proposed Microsnoop [61], a model trained on 10,458 high-quality microscopy images using a masked self-supervised training strategy (see [61] for methodological details). We evaluated it on ten high-quality datasets comprising more than 2.23 million images, covering diverse downstream representation tasks, including protein localization classification, mechanism-of-action prediction, and cell-cycle classification. Using four evaluation metrics (accuracy, Matthews correlation coefficient, balanced accuracy, and the F1-score), Microsnoop demonstrated robust performance and significantly outperformed CytoImageNet and Cytoself. These advances provide useful tools for extracting valuable information from HCS data.

## 3. Applications in Pharmaceutical Research

### 3.1. Deep Learning-Based Intelligent High-Content Screening

**Figure 5:**
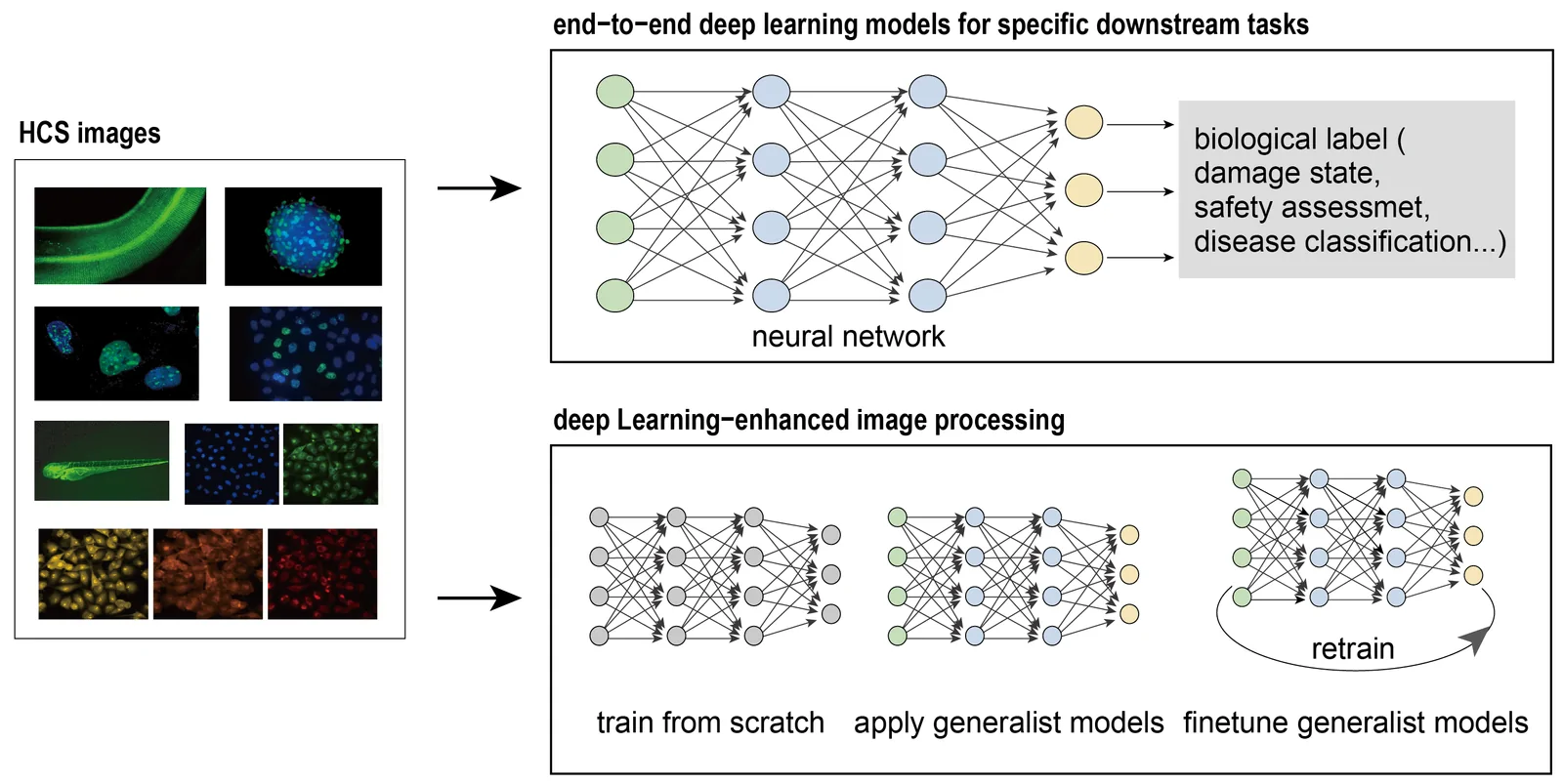
Applications of artificial intelligence in high-content screening.

The rapid development of deep learning has created new opportunities for HCS. These advances are primarily reflected in two aspects (Figure 5). First, HCS images can be directly used to construct end-to-end deep learning models for specific downstream tasks. Second, deep learning-powered cell segmentation and image representation learning can be incorporated into HCS workflows to facilitate single-cell analysis and downstream phenotypic profiling.

One important application is the direct prediction of biological outcomes from HCS images. For example, Grafton et al. [62] established a training dataset using eight compounds with well-characterized mechanisms of action, including bortezomib (a proteasome inhibitor), doxorubicin (a topoisomerase inhibitor), cisapride (a 5-HT4 receptor agonist), and sorafenib (a tyrosine kinase inhibitor). Based on these data, they developed a deep learning model capable of automatically identifying compound-induced cardiotoxicity from cellular images. The proposed approach outperformed conventional methods based solely on fluorescence intensity measurements, demonstrating the ability of deep learning to capture complex phenotypic patterns associated with drug toxicity.

Deep learning has also enabled more sophisticated HCA through accurate cell segmentation and advanced image representation learning. Li et al. [63] developed π-PhenoDrug, a deep learning-based HCS pipeline for phenotype-driven drug discovery. The framework employs a customized UNet++-based nucleus segmentation model (NUSeg), incorporating an Xception encoder and scSE attention modules, to accurately identify individual cells from fluorescence microscopy images. Following segmentation, 106 single-cell morphological features, including intensity, morphology, and texture descriptors, were extracted to construct comprehensive phenotypic profiles. These profiles were subsequently analyzed using both random forest-based supervised classification and unsupervised clustering to evaluate drug-induced phenotypic perturbations and predict compound activity. Applied to melanoma cell lines, π-PhenoDrug successfully identified compounds with potential anti-melanoma effects from an 80-compound library and demonstrated higher sensitivity than conventional single-readout assays in detecting weak but biologically relevant phenotypes. Drug development for disorders such as Parkinson’s disease is often hindered by the lack of robust and screenable cellular phenotypes. To address this challenge, Schiff et al. [64] developed an automated drug discovery platform based on Cell Painting high-content imaging. Using an ImageNet-pretrained Inception network to generate image representations, they analyzed more than one million skin-cell images obtained from 91 individuals, including patients with Parkinson’s disease and healthy controls. Remarkably, this unbiased phenotypic screening strategy, which did not rely on any disease-specific biomarkers or prior biological assumptions, successfully distinguished patients from healthy controls and was even capable of differentiating disease subtypes.

These findings highlight the potential of deep learning-powered HCS to uncover subtle disease-associated phenotypes and facilitate drug discovery in areas where conventional screening approaches are limited. Collectively, these studies demonstrate that deep learning has expanded the scope of HCS from traditional image analysis to comprehensive phenotypic discovery. By enabling end-to-end prediction, accurate single-cell analysis, and biologically meaningful representation learning, deep learning has significantly enhanced the sensitivity and scalability of HCS.

### 3.2. Application in Bioactive Compounds Discovery from Natural Sources

Over the years, HCS has emerged as an important tool in natural product research (Table 1). Xu et al. [65] established an HCS-based phenotypic screening platform to identify TCM compounds capable of inhibiting transforming growth factor-β1-induced epithelial-mesenchymal transition (EMT). A549 cells were stained with TRITC-phalloidin and Hoechst 33342, and high-content imaging was used to quantify multiple morphological features, including cell area, roundness, and length. PCA was subsequently applied to integrate these phenotypic parameters and evaluate EMT-associated morphological changes. Using this strategy, 306 TCM-derived monomeric compounds were screened, leading to the identification of five anti-EMT candidates, namely camptothecin, dimethyl curcumin, artesunate, sinapine, and berberine. Further molecular and functional validation confirmed that these compounds increased E-cadherin expression, reduced vimentin and α-SMA levels, enhanced cell adhesion, and suppressed cell migration, demonstrating the utility of HCS-based phenotypic profiling for natural product discovery.

HCS can even be applied to investigate synergistic effects of different bioactive compounds from natural sources. Li et al. [66] combined HCS with high-resolution mass spectrometry to investigate the antithrombotic mechanisms of Guanxinning Tablet (GXNT), a TCM formula composed of Salvia miltiorrhiza and Ligusticum striatum. Major compounds were characterized using UPLC-HRMS and molecular networking, followed by functional screening in a PHZ-induced zebrafish thrombosis model. The study identified cryptotanshinone and senkyunolide I as two key bioactive compounds that synergistically restored blood circulation and suppressed thrombosis. Mechanistic analyses revealed that cryptotanshinone primarily alleviated oxidative stress and platelet activation, whereas senkyunolide I regulated the coagulation cascade, together producing enhanced antithrombotic efficacy. Chen et al. [67] integrated an angiogenesis-defective zebrafish model with HUVECs to identify pro-angiogenic components from GXNT. Through systematic screening of major herbal components, salvianolic acid B (Sal B) and ferulic acid (FA) were identified as a synergistic compound pair that significantly promoted endothelial cell migration, proliferation, and vascular sprouting. In addition to bioactive compound discovery, HCS has also been applied to safety assessment. For example, Wang et al. [68] established a multiparametric HCS platform for hepatotoxicity assessment of TCM injections. By simultaneously monitoring cellular proliferation, nuclear morphology, mitochondrial function, and membrane permeability in HepG2 cells, the assay rapidly identified potential hepatotoxic formulations and showed strong concordance with subsequent animal toxicity studies.

Recently, an increasing number of researchers have begun to incorporate deep learning into HCS-based natural product research. Chen et al. [33] employed a U-Net-based nuclear segmentation model to isolate individual cells from whole-field microscopy images. Based on prior biological knowledge that the DNA damage response protein 53BP1 forms distinct nuclear foci following ionizing radiation-induced DNA damage, single-cell images were categorized into three classes: damaged, normal, and nonsignaling. Approximately 2000 images were manually annotated for each category and subsequently expanded to 8000 images per class using data augmentation techniques. A VGG-19-based classification model was then trained to predict the damage status of previously unseen compounds. Applying this workflow to a library of 315 natural compounds led to the identification of isoliquiritigenin as a potential DNA damage-protective compound. Xing et al. [69] developed Deep-DPC, which integrates label-free time-series digital phase contrast (DPC) imaging with cellular morphology analysis for anti-fibrotic drug discovery. DPC images were first processed to extract 31 dynamic morphological features, and cells were categorized into six fibrosis-related phenotypic states based on TGF-β-induced morphological changes. Approximately 2000 images were manually annotated for each type and expanded to 48,000 images through data augmentation. An Inception V4-based classifier was subsequently trained to distinguish resting fibroblasts from activated myofibroblasts, achieving 92.3% accuracy on independent test images. Applied to a library of 1400 natural products, Deep-DPC identified 29 anti-fibrotic hit compounds and led to the discovery of Neo-Przewaquinone A, a previously unreported anti-fibrotic molecule that inhibits TGF-β receptor I signaling and alleviates cardiac fibrosis in vivo. Sun et al. [70] developed CPHNet, a deep learning-based Cell Painting screening pipeline for the discovery of anti-high-altitude pulmonary edema (HAPE) agents. The framework first generated more than 100,000 multichannel Cell Painting images from hypoxia-treated alveolar epithelial and pulmonary endothelial cells. A customized YOLOv8-based segmentation network (SegNet) was trained on manually annotated images to accurately identify and segment individual cells and subcellular structures. Subsequently, a six-channel ResNet-50-derived hypoxia scoring network (HypoNet) was trained using over 200,000 single-cell images to quantitatively assess cellular hypoxic states based on morphology. The resulting platform enabled automated phenotypic screening of candidate compounds and successfully identified ferulic acid and resveratrol as promising anti-HAPE agents, whose efficacy was further validated in both a 3D alveolus-on-a-chip model and an in vivo mouse model. Fang et al. [71] developed a high-content phenotypic screening platform based on a single-copy transgenic *Caenorhabditis elegans* strain expressing COL-12::GFP, enabling quantitative visualization of collagen biosynthesis and secretion in vivo. To facilitate automated image analysis, worm images acquired using a high-throughput imaging system were processed with the deep learning-based segmentation tool Scellseg, allowing accurate isolation of individual worms and fluorescence quantification. Using this platform, the authors screened 614 natural small molecules and identified 26 preliminary hits that enhanced collagen-associated fluorescence signals. Subsequent validation demonstrated that Danshensu, Lawsone, and Sanguinarine significantly increased COL-12 protein levels, primarily through post-transcriptional regulation of collagen biogenesis and secretion.

Overall, the integration of deep learning and high-content screening has opened new opportunities for natural product research, and many customized pipelines have been developed for bioactive compound discovery. Some studies [69,71] have begun to explore the use of general-purpose models for data analysis, thereby reducing the need for extensive data annotation and providing new possibilities for the rapid development of discovery pipelines applicable to emerging research scenarios. However, there is still insufficient evidence to demonstrate that performance improvements in segmentation and image representation can directly translate into substantial improvements in hit identification. More research is needed to determine whether advances in AI performance can translate into improved hit identification, biological validation, mechanism-of-action elucidation, and downstream drug discovery outcomes. Otherwise, existing studies have employed multiscale biological models, including whole-organism systems such as *C. elegans*, and combined HCS with cutting-edge technologies such as Cell Painting [72] to improve phenotypic analysis and compound discovery. Nevertheless, most efforts have been limited to the evaluation of single compounds. Given that the therapeutic effects of many natural products arise from multi-component synergy, extending deep learning-enabled HCS to characterize synergistic interactions and combinatorial effects remains a major challenge and a promising avenue for future investigation. Last but not least, because many natural products can induce strong stress or cytotoxic phenotypes, considering the safety of natural products during the screening process, such as using HCA to distinguish specific pharmacological responses from nonspecific cellular stress or cell death, is also an important research direction.

## 4. Conclusions and Perspectives

Artificial intelligence has transformed high-content screening, enabling more accurate image analysis, robust phenotypic profiling, and efficient bioactive compound discovery. By integrating advances in deep learning, cell segmentation, and feature extraction, HCS has evolved from a conventional image-analysis technique into a useful platform for phenotype-driven drug discovery. The complexity of chemical structures and biological mechanisms often limits the effectiveness of traditional target-based screening approaches. These approaches include not only single-target screening but also multi-target strategies, which commonly rely on computational methods to estimate the potential interactions or binding affinities between compounds and predefined targets [73,74]. Such approaches generally require prior knowledge of the targets of interest or a drug–target network database, making it challenging to identify unexpected or previously unknown targets. In contrast, AI-HCS provides a complementary phenotype-driven strategy that can rapidly capture complex cellular phenotypes (such as Cell Painting) resulting from the unknown modulation of multiple targets and signaling pathways, without requiring the targets to be specified in advance. This feature is particularly relevant to natural products, whose biological activities often arise from multi-target and multi-pathway interactions. Compared with existing multi-target approaches outside the HCS framework, HCS therefore offers a complementary strategy for investigating the phenotypic consequences of complex biological mechanisms and may facilitate the discovery of previously unrecognized mechanisms of action. Despite these advances, several challenges remain before AI-powered HCS can realize its full potential in natural product-based drug discovery.

One of the major limitations currently hindering the development of AI-driven HCS is the lack of large-scale, standardized datasets specifically designed for natural product research. In contrast to the computer vision field, where publicly available datasets such as ImageNet [75,76] have played a pivotal role in advancing deep learning, phenotypic datasets for natural products remain fragmented across individual laboratories and research projects. Most published studies focus on relatively small collections of compounds, making it difficult to train robust and generalizable AI models. Future efforts should focus on establishing comprehensive natural product phenotypic databases that integrate high-content imaging data, chemical structures, biological activities, molecular targets, and experimental metadata. This can help mitigate the risk of data leakage during model training, such as when data from the same batch, or even from the same plate or well, are included in both the training and evaluation sets. Such leakage may introduce model bias or overfitting and compromise the reliability of performance evaluation. Additionally, metadata such as drug concentration and treatment duration can also be incorporated to explore concentration-dependent and time-dependent phenotypes. Establishing standardized datasets would therefore help promote more reliable benchmark evaluation and facilitate meaningful cross-study comparisons. Furthermore, standardized data formats [77] and annotation protocols will be essential for improving data interoperability and facilitating the development and dissemination of reusable analytical pipelines.

Current AI applications in HCS are primarily concentrated in image analysis tasks, such as cell segmentation, feature extraction, and phenotype classification. However, the entire HCS workflow contains numerous stages that could benefit from artificial intelligence, such as image acquisition, quality control, hit identification, and lead prioritization. Recent advances in multimodal learning [78] and large language models [79] provide new opportunities to develop end-to-end intelligent screening systems. In the future, AI is expected to evolve from a data-analysis tool into a comprehensive decision-support system that assists researchers throughout the entire drug discovery process.

Another important future direction is the construction of intelligent and digitalized HCS platforms. Current screening workflows often involve multiple disconnected software packages and substantial manual intervention, creating bottlenecks in data processing, interpretation, and knowledge extraction. The integration of automated microscopy, laboratory robotics, cloud computing, artificial intelligence, and laboratory information management systems has the potential to establish fully digitalized screening environments. Such platforms [80,81] could enable automated experiment execution, real-time image analysis, dynamic decision-making, and closed-loop optimization. For natural product research, intelligent HCS platforms could further integrate chemical profiling technologies such as LC-MS, metabolomics, and molecular networking, creating a unified framework that links chemical composition, cellular phenotypes, and biological activities.

In conclusion, the convergence of artificial intelligence, high-content screening, and natural product science is opening new avenues for phenotype-driven drug discovery. The establishment of large-scale phenotypic databases, the integration of AI throughout the screening workflow, and the development of intelligent digitalized platforms are expected to accelerate the discovery of bioactive compounds from natural sources and promote the next generation of natural product-based pharmaceutical innovation.

## Figures and Tables

**Table 1:** Representative applications of HCA in natural product research.

Perturbation Reagent (Number)	Screening Model	HCA Method	Role of the AI Method	Application	Reference
Natural compound library (306)	A549 cell	Manual features (cell area, cell roundness, cell length, and cell number)	N/A	Epithelial–mesenchymal transition-related diseases	[65]
TCM and its components	zebrafish	Manual features (platelet circulation, number of intersegmental vessels)	N/A	cardiovascular diseases	[66,67]
TCM (243)	HepG2 cell	Manual features (cell number, nuclear area, mitochondrial mass, mitochondrial membrane potential, and plasma membrane permeability)	N/A	drug-induced liver injury	[68]
Natural compound library (315)	HeLa cell	FociNet, U-Net, VGG-19	cell segmentation and classification	DNA damage-related diseases	[33]
Natural compound library (1400)	NIH-3T3 cell	Deep-DPC, Inception V4	classification	fibrosis	[69]
Natural compound library (11)	A549 and HPMEC	CPHNet, SegNet, HypoNet	cell segmentation and classification	high-altitude pulmonary edema (HAPE)	[70]
Natural compound library (614)	*C. elegans*	Scellseg	cell segmentation	collagen-related diseases	[71]

## Data Availability

No new data were created or analyzed in this study. Data sharing is not applicable to this article.
